# Whole genome sequencing and identification of *Bacillus endophyticus* and *B. anthracis* isolated from anthrax outbreaks in South Africa

**DOI:** 10.1186/s12866-018-1205-9

**Published:** 2018-07-09

**Authors:** Kgaugelo Edward Lekota, Oliver Keoagile Ignatius Bezuidt, Joseph Mafofo, Jasper Rees, Farai Catherine Muchadeyi, Evelyn Madoroba, Henriette van Heerden

**Affiliations:** 1 The Biotechnology Platform, Agricultural Research Council, Private Bag X5, Onderstepoort, 0110 South Africa; 2 Department of Veterinary Tropical Diseases, University of Pretoria, Private bag X4, Onderstepoort, 0110 South Africa; 3 Bacteriology section, Agricultural Research Council-Onderstepoort Veterinary Institute, Private Bag X5, Onderstepoort, 0110 South Africa; 4 College of Agriculture and Environmental Sciences, University of South Africa, Florida Campus, Christiaan De Wet/ Pioneer Dr, P.O. Box X6, Florida, 1710 South Africa; 5 Department of Biochemistry and Microbiology, University of Zululand, Private Bag X1001, KwaDlangezwa, 3886 South Africa

**Keywords:** *Bacillus endophyticus*, *Bacillus anthracis*, Poly-γ-glutamic acid (PGA), Whole genome sequencing (WGS)

## Abstract

**Background:**

*Bacillus endophyticus* is a soil plant-endophytic bacterium, while *B. anthracis* is the causative agent of anthrax. The virulence factors of *B. anthracis* are the plasmid encoded tripartite toxins (pXO1) and poly-γ-glutamic acid (PGA) capsule (pXO2). *B. endophyticus* isolated alongside *B. anthracis* from animals that died of anthrax in Northern Cape Province (NCP), South Africa, harbored polyglutamate genes. The study compared the characteristics of *B. anthracis* and *B. endophyticus* with other *Bacillus* species with a focus on the presence of the PGA capsule or/and unbound PGA. The morphology and whole genome sequence analysis of *B. endophyticus* strains and *B. anthracis* were compared.

**Results:**

In conventional microbiology, *B. endophyticus* showed gram-positive round-shaped rods in single/short chains, which were endospore-forming, non-motile, non-haemolytic with white and dry colonies, and γ-phage resistant. *B. anthracis* was differentiated from *B. endophyticus* based on the latter’s box-shaped rods in pairs/long chains, white-grey and slimy colonies, encapsulated and γ-phage susceptible. The study identified a PGA polyglutamate synthase operon that consisted of *pgs*BCA, γ-glutamyltranspeptidase (*ggt*) and *pgs*E in *B. endophyticus* genomes.

**Conclusions:**

PGA regions of *B. anthracis* contain *cap*BCADE genes located in the pXO2 required for capsulation formation, while *B. endophyticus* contain the *pgs*BCAE genes in the chromosome. Whole genome and microbiology analysis identified *B. endophyticus*, as a non-capsuled endospore-forming bacterium that consists of PGA required for biosynthesis. *B. endophyticus* strains do not synthesize surface associated PGA, therefore capsule visualization of *B. anthracis* is a key diagnostic characteristic. The study highlights the significance of using whole genome shotgun sequencing to identify virulence and other important genes that might be present amongst unknown samples from natural outbreaks. None of the *B. anthracis* related plasmids or virulence genes were found in the *B. endophyticus* genomes.

**Electronic supplementary material:**

The online version of this article (10.1186/s12866-018-1205-9) contains supplementary material, which is available to authorized users.

## Background

*Bacillus endophyticus* is regarded as a plant-endophytic bacterium that is found in the inner tissues of plants, specifically cotton [1]. It is present either as gram-positive single rod-shaped cells or as chains that can be short or long, non-haemolytic and non-motile. Biochemical characteristics that differentiate *B. endophyticus* from other *Bacillus* species include the inability to reduce nitrate (NO^3−^) to nitrite, casein and starch, as well as, ampicillin and NaCl resistance [1].

*B. anthracis* is the causative agent of anthrax, and primarily affects herbivorous animals, although all mammals can also be affected. The vegetative cells of *B. anthracis* appear ‘box-shaped’ either in pairs or chains. It is phenotypically characterized as gram-positive aerobic rods (3–5 μm × 1 μm), that are non-haemolytic, non-motile, penicillin and γ-phage resistant [2]. However, it is distinguishable from its close relatives by its ability to synthesize virulence factors encoded on plasmids, pXO1 and pXO2. The pXO1 (182 kb) contains genes encoding for the tripartite anthrax toxins (protective antigen, lethal factor and edema factor) and pXO2 (96 kb) encodes a five-gene operon *cap*BCADE (capsule biosynthesis genes), which synthesizes a poly-γ-glutamic acid (PGA) capsule [3, 4]. Capsule biosynthesis genes are transcribed as a single operon predicted to encode proteins for the biosynthesis, transport and attachment of D-glutamatic acid residue on the bacterial surface [5]. The anthrax capsule activators (*acp*A and *acp*B) located on pXO2 are controlled by anthrax toxin activator (*atx*A) located on pXO1 [5]. The PGA capsule enables host immune system evasion by protecting the vegetative cells from phagocytosis by macrophages [5]. The vegetative cells of *B. anthracis* have also been shown to secrete the PGA capsules under anaerobic conditions and in the presence of bicarbonate [3, 5].

Many pathogenic bacteria require a cell-associated capsule for virulence [6]. Capsule composition of bacteria can be in a form of polypeptide (poly-glutamate) or polysaccharide. Poly-γ-glutamic acid (PGA) is a poly-anionic polymer that may be composed of only D, only L or both glutamate enantiomers [4, 7]. Most strains producing PGA are members of the gram-positive *Bacillus* group. The function of PGA depends on whether it is bound to peptidoglycan or unbound/released. In the bound state it forms the capsule, whereas in the secreted/unbound state it is released into the environment [4, 8]. The uncommon bound PGA capsule only includes *B. anthracis* and *Staphylococcus epidermidis* that synthesize the anchored (surface-associated) PGA, which enables them to act as a virulence factor [4]. The *B. anthracis* PGA synthesis genes are encoded on the pXO2 consisting of *cap*B*, cap*C*, cap*A and *cap*E, while *cap*D acts as the peptidoglycan binding/anchoring site [4, 7, 9]. The corresponding polyglutamate biosynthesis pathway orthologs in *B. subtilis* includes *pgs*B*, pgs*C and *pgs*AA [10] and *pgs*S has been suggested to induce the release of PGA [4, 7]. The *cap*BCADE genes of *B. anthracis* encoded on pXO2 have functional orthologs encoded on the chromosomes of *B. subtillis/licheniformis* and other *Bacillus* species [4, 11]. Few species such as *B. anthracis* and *S. epidermidis* have been reported to produce the PGA capsule [4]. The unbound PGA have been reported to *Bacillus* species such as *B. cereus* strains ATCC 10987, 14,579 and *B. thuringiensis* 97–27, AI Hakam [4]. *B. cereus* biovar *anthracis* strains isolated from great apes that died of anthrax symptoms in west and central Africa were shown to harbor the *B. cereus* chromosome and pXO2-like plasmid [12] that contained the PGA capsule genes identical to those of *B. anthracis*.

Gene sequences that encode for the formation of PGA and capsules on the pathogenic and non-pathogeneic species need to be compared and distinguished from their close relatives [11]. This is essential especially when some of the virulence gene sequences and morphological characteristics are used for identification and diagnosis of anthrax. In this study, *B. endophyticus* strains were isolated alongside *B. anthracis* strains from animals that died of anthrax in Northern Cape Province (NCP), South Africa in an outbreak that occurred in 2009. *B. endophyticus* is regarded a plant-endophyte and it is uncommon to be isolated from blood or animals. The *B. endophyticus* strains that were isolated alongside *B. anthracis* strains had some of the similar morphological, biochemical and some genetic characteristics compared to the anthrax causing bacteria. In our previous study, conventional PCR detected PGA gene regions in both *B. anthracis* and *B. endophyticus* isolates and attempts were made to distinguish and identify these strains using routine and non-routine diagnostic methods [13]. The *B. endophyticus* strains were identified using non-routine diagnostic Omnilog (Biolog) and 16S rRNA sequencing methods and differentiated based on routine diagnostic microbiological tests and real time PCR. Therefore, in order to enhance and contribute to the unequivocal diagnosis of *B. anthracis*, the goal of this study was to perform comparative analysis of the *B. endophyticus* and *B. anthracis* strains from the afore-mentioned outbreak as well as contribute towards the scant genome information of *B. endophyticus*. Thus the virulence genes of *B. anthracis* occurring on the plasmids were investigated, as well as the capsule and phenotypic characteristic of related *Bacillus* species were summarized using results from this study and published literature to enhance and contribute towards anthrax diagnosis.

## Results

### Phenotypic characterization

*B. endophyticus* strains reported in the study were isolated from the environment and/or animals that died of *B. anthracis* during the 2009 anthrax outbreak in Northern Cape Province (NCP) (Table 1). On sheep blood tryptose agar (SBTA) at 5% CO_2_, colonies of *B. anthracis* appeared whitish-grey, smooth, dry and shiny (medusa head), while *B. endophyticus* colonies were circular white, slimy or rough (Additional file 1: Figure S1 (2)). The *B. endophyticus* colonies on nutrient agar supplemented with 0.8% sodium bicarbonate at 5% CO_2_ were smaller and circular, non-mucoid and wet (Additional file 1: Figure S1A), whereas *B. anthracis* colonies appeared circular, mucoid and shiny (Additional file 1: Figure S1B). Colony morphology of the *B. endophyticus* strains was observed after 24 h in culture compared to *B. anthracis*, which was observed earlier (12–24 h) on sodium bicarbonate supplemented nutrient agar.

**Table 1:** *Bacillus endophyticus* and *B. anthracis* strains isolated from animal anthrax cases in Northern Cape province (NCP) in South Africa

Strain number	Animal source^c^	Specimen	Isolation date	Provinces and location (farm or town) in South Africa	*Bacillus* species
3617_2C^a^	*Tragelaphus strepsiceros* (Kudu 1)	Ear blood	May-09	NCP (Klipfontein)	*B. endophyticus*
3618_1C^a^	*Tragelaphus strepsiceros* (Kudu 2)	Ear blood	May-09	NCP (Kimberly)	*B. endophyticus*
3631_9D^a^	*Ovis aries* (Sheep 1)	Ear blood	May-09	NCP (Kimberly)	*B. endophyticus*
3631_10C^a^	*Ovis aries* (Sheep 2)	Ear blood	May-09	NCP (Kimberly)	*B. endophyticus*
3618_2D	*Tragelaphus strepsiceros* (Kudu 2)	Soil	May-09	NCP (Klipfontein)	*B. anthracis*
3631_1C^b^	*Tragelaphus strepsiceros* (Kudu 3)	Ear blood	May-09	NCP (Klipfontein)	*B. anthracis*
3617_1C	*Tragelaphus strepsiceros* (Kudu 1)	Ear blood	May-09	NCP (Klipfontein)	*B. anthracis*

^a^The strains that were subjected for sequencing

^b^Draft genome from previous study [14]

^c^Kudu 1, kudu 2, sheep 1 etc. refer to different animal carcasses with *B. endophyticus* (3617_2C) and *B. anthracis* (3617_1C) isolated from the same kudu carcass (kudu 1)

**Fig. 1:**
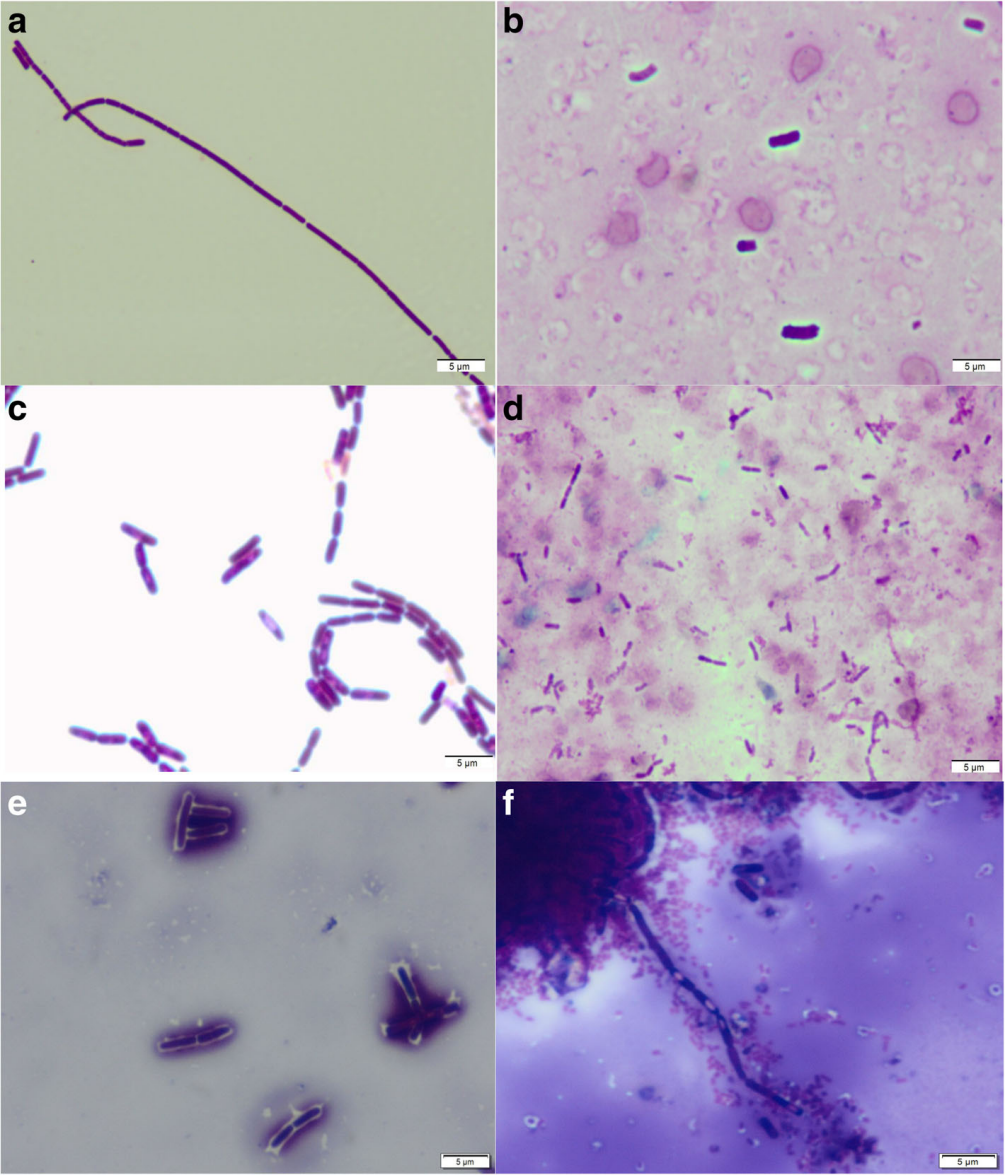
Phenotypic electron microscopic examination of the morphology of *Bacillus anthracis* and *B. endophyticus* strains. (**a)** Gram-positive vegetative cells of *B. anthracis* Sterne long, bacilli chains, (**b**) *B. anthracis* 3618_2D capsulated in blood serum, (**c**) Gram-positive vegetative cells of *B. endophyticus* short, bacilli chains, and (**d**) *B. endophyticus* 3631_9D non-capsulated in blood, (**e**) capsulated *B. anthracis* 3618_2D and (**f**) *B. endophyticus* 3631_9D non-capsulated with spores after incubation on nutrient agar containing 0.8% sodium bicarbonate in the presence of 5% CO_2_

Gram-positive *B. anthracis* cells occurred in box-shaped rods in pairs and/or long chain rods (Fig. 1a) that are encapsulated (cap^+^) after incubation at 5% CO_2_ in blood (Fig. 1b), while the gram-positive *B. endophyticus* appeared as round-edged rods either as single and/ or short chains (Fig. 1c, Table 2). No capsules were observed in *B. endophyticus* strains after incubating at 5% CO_2_ (Fig. 1d). *B. anthracis* 3631_1C [14] and *B. anthracis* Sterne strains were non-capsulated (cap^−^) since they lack pXO2 while *B. anthracis* 20SD was capsulated (Fig. 1e). The terminal ellipsoidal spores were also observed in *B. endophyticus* 3631_9D strain using the copper sulphate stain after 24 h incubation on nutrient agar containing 0.8% sodium bicarbonate (Fig. 1 and Additional file 2: Figure S2A-D).

**Table 2:** Comparison of phenotypic and biochemical characteristics of *Bacillus endophyticus, B. anthracis, B. cereus, B. megaterium* and *B. smithii*

Microbiological characteristics	*B. endophyticus*	*B. anthracis*	*B. cereus*	*B. megaterium*	*B. smithii*
Gram reaction	+	+	+	+	+
Rods (μm)	2.5–3.5 × 0.5–1.5	3–6 × 1–1.25	3-5 × 1–1.2	2–5 × 1.2–1.5	5–6 × 0.8–1.0
Spores	Ellipsoidal/cylindrical	Ellipsoidal/cylindrical	Ellipsoidal/cylindrical	Ellipsoidal/Spherical	Ellipsoidal/cylindical
Haemolysis	NH	NH	H	H^%%^	ND
Motility	Non-motile	Non-motile	Motile	Non-motile	motile
Capsule	–	+	–	+	–
Penicillin	S	S	R	ND	ND
Gelatin hydrolysis	–	+ ^*^	+	+	–
Nitrate reduction	–	+	+	(−)	–
Starch hydrolysis	–	+	+	+	W+
Voges Proskaeur (VP)	–	+	+	–	–
γ –phage	R	S^$^	R	S^@^	ND
Casein	–	+	+	+	–
Egg yolk/Lecithinase	–	+	+	–	–
Urea hydrolysis	–	–	–	-^%%^	–
Citrate	W+	–	+	+	(−)
NaCl	2–10%	5%	2–7%	7%	2–3%
Catalase	+	+	+	+	+
Indole	–	–	–	–	–
Oxidase	+	+	–	(+)	+

Abbreviations: +, positive, W+, weakly positive, −, negative, (), variable, S, susceptible, R, resistance, ND, not determined, NH, non-haemolytic, ^*^, inverted fir tree, ^$^, *B. anthracis* strains resistance to γ-phage have been reported [22], ^@^ resistant γ-phage *B. megaterium* strain has been reported at Kansas State University [44] . All data of *B. endophyticus* were obtained in the study and supplemented with information of Reva et al. [1]. *B. anthracis* and *B. cereus* information was compiled from WHO [42]; ^%%^ Beesley et al. [27] reported *B. megaterium* non-haemolytic and urea hydrolysis strains

The comparison of phenotypic properties of *B. endophyticus, B. anthracis, B. cereus B. megaterium* and *B. smithii* strains is shown in Table 2. *B. anthracis* and *B. cereus* were compared in Table 2 as they belong to *B. cereus sensu lato* group, while *B. megaterium* is closely related to *B. endophyticus* based on whole genome sequence and some of the microbiological features are similar to *B. anthracis*. *B. smithii* is a closely related species of *B. endophyticus* based on 16S rRNA sequence gene. However, Table 2 shows that *B. cereus* and *B. smithii* are both motile and can easily be excluded from *B. anthracis*. *B endophyticus* is a gram-positive, non-capsulated, non-motile, round-edged rod that is endospore-forming, non-hemolytic, penicillin sensitive but γ-phage resistant bacterium. *B. anthracis* is a gram-positive capsulated, non-motile, box-shaped rod that is endospore-forming, non-hemolytic, penicillin and γ-phage sensitive (Table 2). In this study biochemical characterization showed some common results between *B. anthracis* and *B. endophyticus* including the positive reaction for catalase and oxidase and negative reaction for indole (Table 2). Biochemical properties of *B. endophyticus* that differentiated it from other *Bacillus* species included inability to reduce nitrate to nitrite, hydrolyze casein, gelatin, and starch, as well as resistance to NaCl. The absence of lecithinase and Voges Proskaeur (VP) can be used to distinguish *B. endophyticus* from *B. anthracis* (Table 2)*.*

### 16S rRNA gene phylogenetic analysis

The 16S rRNA gene sequences of *B. endophyticus* strains 3631_9D, 3617_2C, 3631_10C and 3618_1C strains were used to mine for other 16S rRNA gene sequences through BLAST homology searches. The sequenced *B. endophyticus* strains 3631_9D, 3617_2C and 3631_10C showed a 100% similarity with the 16S rRNA gene sequences of *B. endophyticus* strains (A6, S160(2), 2DT and uncultured bacterium 12TR2ACLN347) (Additional file 3: Figure S3). Strain 3618_1C grouped with majority of the uncultured bacterium (12TRACLN435 and 12TRACLN431) obtained from NCBI. The *B. cereus* sensu *lato* group grouped separately from the *B. endophyticus* based on 16S rRNA gene region (Additional file 3: Figure S3)*.*

### Average nucleotide identities, pan-genome analyses functional classification of orthologous genes

**Fig. 2:**
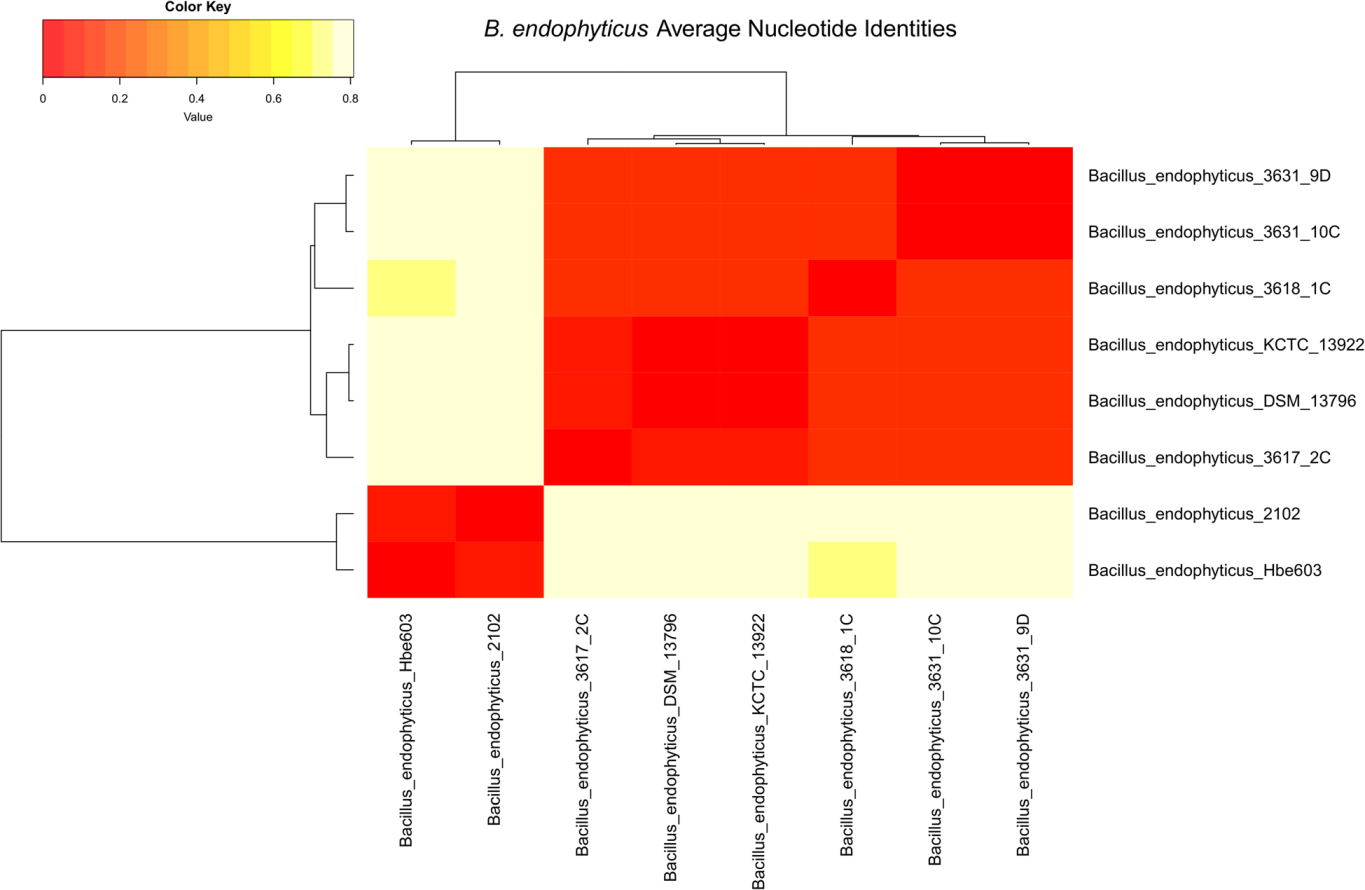
ANI - A heatmap representing the degree of similarity shared among the 8 *Bacillus endophytiucs* isolates based on the average nucleotide identities of their coding domain sequences (CDSs). The heatmap was derived from an average nucleotide identity matrix determined from the high (dark orange) to low (light yellow) similarities of CDSs derived from the *B. endophyticus* genomes

South African *B. endophyticus* sequences (3617_2C, 3618_1C, 3631_9D, 3631_10C) had a total of approximately 5.1 to 45.3 million reads with an average length of 94 nucleotides after trimming. Sequenced reads were de novo assembled (Table 3) and annotated using PGAAP for further classification of the *B. endophyticus* strains. The heat map (Fig. 4) indicated the average nucleotide identities of the *B. endophyticus* CDSs of South African sequenced strains and available whole genome sequences (2102, Hbe603, A6, S160(2), 2DT, KCC 13922, DSM13796 and uncultured bacterium 12TR2ACLN347). The sequenced *B. endophyticus* strains in this study as well as *B. endophyticus* DSM 13976 and KCTC 13922 had the same profile (with an ANI score of > 98%); *B. endophyticus* 3617_2C is highly related with this two genomes forming their own sub-clade, but clustered separate from *B. endophyticus* 2102 and Hbe603 strains (Fig. 2). *B. endophyticus* 3618_1C grouped separately amongst the sequenced *B. endophyticus* strains.

**Table 3:** Genome comparison features of the *Bacillus endophyticus* strains used in the study

	*B. endophyticus* 3631_9D	*B. endophyticus* 3618_1C	*B. endophyticus* 3631_10C	*B. endophyticus* 3617_2C	*B. endophyticus* Hbe603^a^
Genome size (bp)	5,311,808	5,379,838	5,243,706	5,319,031	5,313,985
GC content (%)	36	36	36	36	36
Total contigs	57	68	60	99	9
N50	304,287	152,600	277,642	99,724	4,865,574
Maximum contig	820,841	657,546	874,718	224,909	NA
Minimum contig	307	141	1164	227	NA
Coding sequences (CDS)^b^	5310	5470	5358	5408	5455
RNAs	68	54	50	47	114
Prophage regions	1	7	2	2	4
Chromosome (size)	5,056,260	5,011,594	5,305,974	5,231,075	4,865,574
Number of plasmids^c^	7	4	6	6	8

^a^*Bacillus endophyticus* Hbe603 was used as a reference strain in this study

^b^Coding sequences predicted using RAST

^c^BLASTn with an e-value of 1e-10 and > 90% identity was used against the *B. endophyticus* Hbe603 plasmids

*NA* not available

**Fig. 3:**
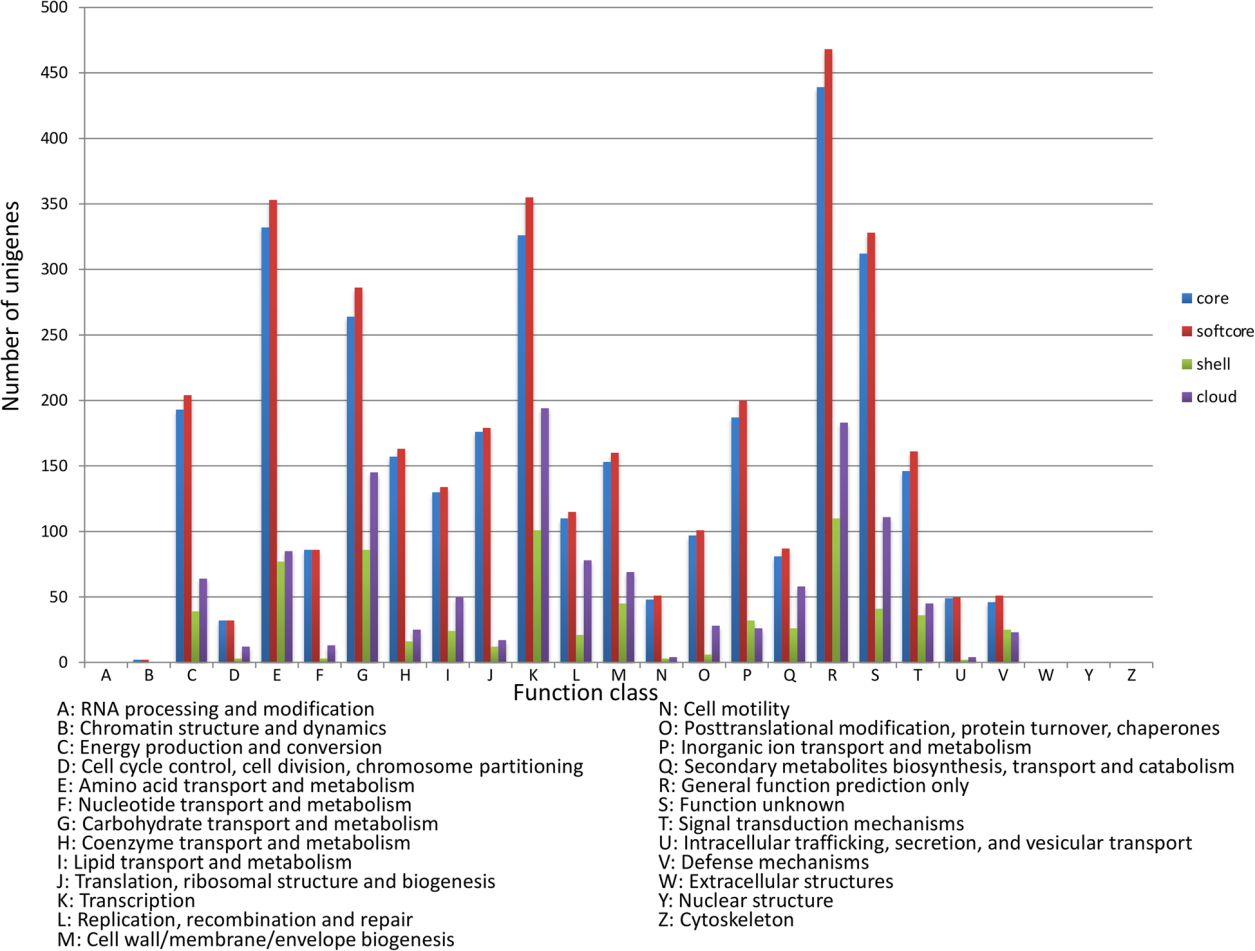
COG - Clusters of orthologous group (COG) analysis of the *Bacillus endophyticus* pan-genome. Each bar corresponds to the four different pan-genome compartments, whereas their heights correspond to the total number of genes in the compartments that were assigned to the COG functional categories

The pan-genome homology analysis of 4 South African *B. endophyticus* and Hbe603 strains identified 7154 clusters of protein coding genes with 3711, 3954, 997 and 2203 clusters represented the core, softcore, shell and cloud genomes, respectively (Fig. 3). In this study the *B. endophyticus* has more genes assigned to the core than the accessory genes (shell and cloud clusters), but the latter might increase when more genomes are sequenced and become available (Fig. 3). In the COG category assignments, the core and the accessory genomes have a slightly different number of genes assigned to the defense mechanisms category (Fig. 3 category V) as in most cases this category is mainly abundant in the accessory genome [15]. The core cluster dominates all the other categories, including categories for function unknown (S) and general prediction only (R) in Fig. 3.

### Genomic features of *B. endophyticus* strains

Comparative genomics of the draft sequenced *B. endophyticus* strains in the study and complete genome *B. endophyticus* Hbe603 showed almost equivalent genome sizes with the complete genome of *B. endophyticus* Hbe603 (Table 3). The GC content of the sequenced *B. endophyticus* genomes are approximately 36%, and similar to the *B. endophyticus* Hbe603 and other *B. endophyticus* strains used in pan-genome analysis. The complete genome of Hbe603 is 5.31 Mb and consists of a chromosome and 8 plasmids [16]. Annotation using RAST [17], predicted the number of coding sequences of *B. endophyticus* Hbe603 to be 5455 that is slight higher than the sequenced genomes in this study except for 3618_1C. High numbers of accessory genes of *B. endophyticus* 3618_1C are represented in the unknown function or as hypothetical proteins. A total of 5310, 5431, 5358, and 5408 predicted coding sequences in strains 3631_9D, 3618_1C, 3631_10C and 3617_2C respectively (Table 3). RAST analyses showed *B. megaterium* DSM 319 to be the closest neighbor to the *B. endophyticus* strains with the comparative analysis using sequence similarity option.

### Plasmids of *B.endophyticus*

*B. endophyticus* Hbe603 complete genomes consist of 8 plasmids. The roles of the plasmids have never been reported in *B. endophyticus* Hbe603 strain. The draft genomes of *B. endophyticus* strains sequenced in this study each presented 4–7 plasmids (Table 3, Additional file 4: Table S1). Comparative analysis of the sequenced *B. endophyticus* strains with *B. endophyticus* Hbe603 consisted of partial regions of plasmids, while pBEH1, pBEH6, and pBEH7 are the common plasmids shared. Plasmids sizes of draft genome *B. endophyticus* strains were significant smaller than the *B. endophyticus* Hbe603 plasmids (Additional file 4: Table S1). None of the *B. endophyticus* plasmids were similar with the *B. anthracis* pXO1 and pXO2 plasmids.

### Virulence, resistance and defense genes

Coding sequences linked to antibiotic- and toxic compound resistances were identified in the *B. endophyticus* strains. Comparative analysis of *B. endophyticus* 3618_1C, 3631_9D, 3631_10C, KCTC 13922, and DSM 13796 showed unique coding sequences that include arsenical-resistance protein *Acr3*, copper resistance protein D for copper homeostasis, multidrug resistance transporter *Bc*r/*Cfl*A family and fosfomycin resistance protein *fos*B that are absent in the *B. endophyticus* Hbe603 and 2102 genomes. *B. endophyticus* 3617_2C strain also contained these coding sequences except the *Acr3* and the multidrug resistance transporter *Bc*r/*CflA* family CDS. The transcriptional regulator *Nfx*B was present in *B. endophyticus* 3618_1C and 3617_2C strains (i.e. absent in the other compared *B. endophyticus* strains in this study). This transcriptional regulator is involved in the MexC-MexD-OprJ multidrug efflux system that contributes to antibiotic- or toxic compounds resistances [18]. The genome analyses of *B. endophyticus* strains confirmed the presence of CDS for the macrolide-specific efflux protein *mac*A and permease protein *macB* for the multidrug resistance efflux pumps, except in strains 3618_1C and 2102. The *MacAB-TolC* macrolide efflux transport system has mostly been studied in gram-negative bacteria. The presence of *mac*A in the system is known to stimulate the ATPase activity of *macB* to bind macrolides such as erythromycin and azithromycin. Meanwhile the overproduction of *mac*A and *mac*B results in an increase resistance to the macrolides antibiotics [19]. *B. endophyticus* is regarded as plant-endophytic bacterium that survives high-salt concentration [1, 13]. The sigma-M predicted to response to high concentration of salt [20] was found in the 8 compared *B. endophytcicus* genomes in this study. Jia et al. [16] predicted other sigma factors responsible for gene regulation in *B. endophyticus*.

### Bacillus endophyticus prophages

PHAGE_Bacill_phBC6A52 was the common intact prophage in strains 3631_9D and 3631_10C. *B. endophyticus* 3631_10C presented additional two partial prophage regions annotated as PHAGE_Lister_B054_NC_009812 and Bacill_1_NC_009737. The latter, PHAGE_Bacill_1_NC_009737, was also present in *B. endophyticus* 3617_2C. About 7 prophage regions were identified in *B. endophyticus* 3618_1C strain (Table 3). This included PHAGE_Bacill_G_NC_023719, PHAGE_Burkho_phi023719, PHAGE_Synech_S_MbCM100_NC_023584, PHAGE_Entero_phi92_NC_023693, PHAGE_Escher_vB_EcoM_UFV13_NC_031103, PHAGE_Bacill_SP_15_NC_031245 and PHAGE_Bacil_BM5_NC_029069. The 7 prophages were also identified in *B. endophyticus* DSM_13,796 and KCTC 13922 except for PHAGE_Entero_phi92_NC_023693 and PHAGE_Escher_vB_EcoM_UFV13_NC_031103. However, the prophage regions differ in their sizes. Only 4 prophages were determined in the *B. endophyticus* Hbe603 reference strain, whereby most were annotated as hypothetical proteins [16]. In *B. endophyticus* 2102, no prophage sequence regions were identified. Comparative analysis of prophages between *B. anthracis* strains 3631_1C and 20SD [14] and *B. endophyticus* sequenced in this study indicated that the four Lambda Ba prophages remain unique to *B. anthracis*.

### PGA biosynthesis complex

**Fig. 4:**
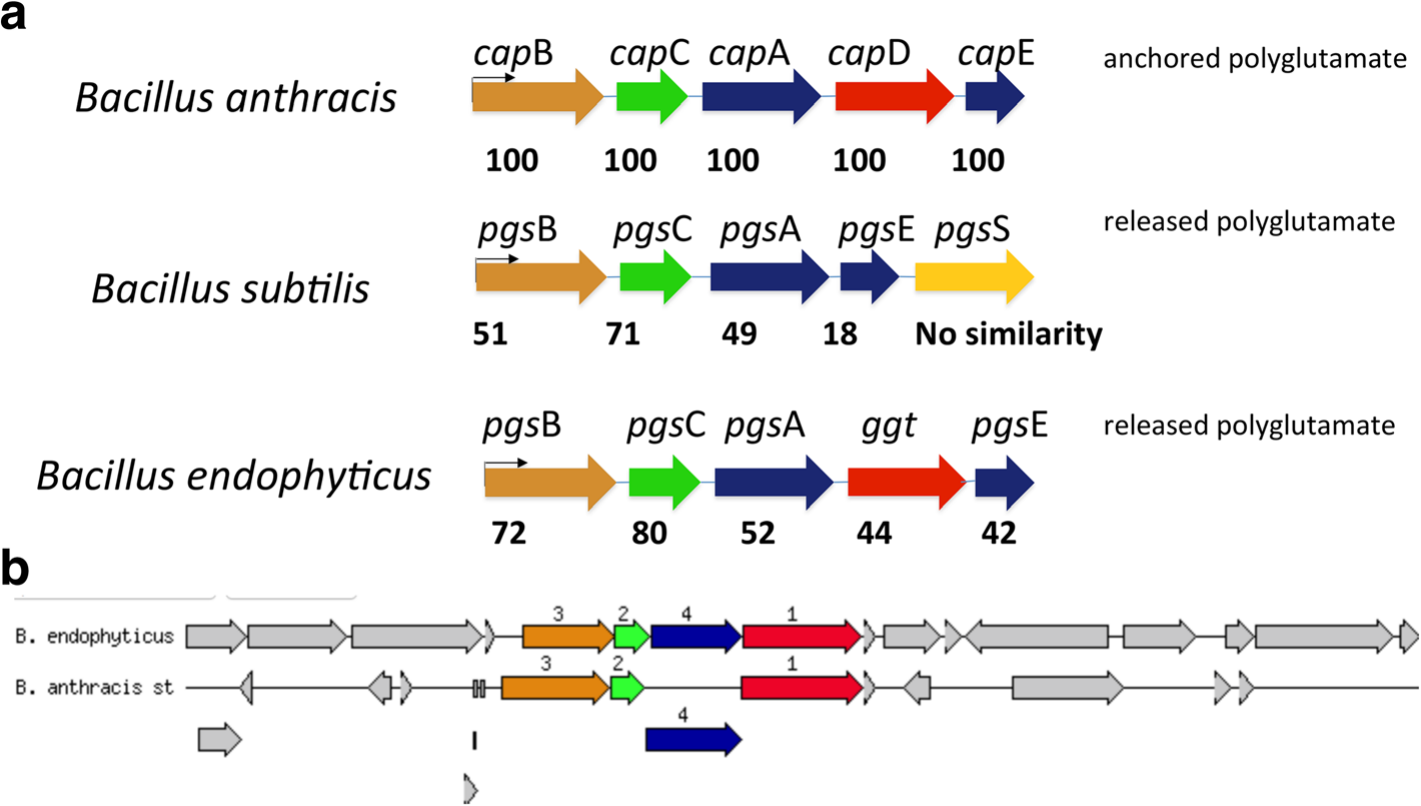
Comparative structure of the polyglutamate (PGA) subunit genes of the *Bacillus endophyticus* 3631_9D, *B. anthracis* Ames and *B. subtilis* natto IF03336. All *cap*/*pgs* coding sequence are indicated in colours with (**a**) representing the comparison of the PGA synthetic operon of *B. anthracis*, *B. subtilis*, and *B. endophyticus*. Numbers indicates amino acid sequence identities (%) of the cap/pgs proteins to those of *B. anthracis*. (**b**) Indicates the annotated sequence based comparison of the *B. endophyticus* 3631_9D and *B. anthracis* Ames PGA genes. Number 1 (red) represents *pgs*/*cap*D, 2 *pgs/cap*C, 3 (brown) *pgs/cap*B, 4 (blue) pgs/*cap*A

The PGA subunits *pgs*B, *pgs*C, *pgs*A and γ-glutamyl transpeptidase (*ggt*), and *pgs*E genes were present in the 4 sequenced *B. endophyticus* strains (3617_2C, 3618_1C, 3631_9D, 3631_10C) and other 4 compared *B. endophyticus* genomes (2102, Hbe603, KCC 13922, DSM 13796) in this study. The PGA subunits of *B. endophyticus* genomes are located in the chromosome compared to the plasmid, pXO2, of *B. anthracis*. In *B. anthracis*, the PGA subunits are presented and annotated as *cap*BCADE (Fig. 4). They are associated with the synthesis of the poly-γ-glutamate capsule formation rather than a released PGA. Due to no capsule formation observed in the *B. endophyticus* strains, this suggests that PGA biosynthesis is associated in a released form. *Bacillus* species genomes i.e. *B. subtilis*, and *B. licheniformis* (Fig. 4) consist of pgs subunits. The amino acid sequence identities of *cap*/*pgs* subunits to *B. anthracis* are indicated in Fig. 4 indicating the percentages of amino acid similarities between *B. endophyticus, B. anthracis* and *B. subtilis*. *B. endophyticus* and *B. subtilis* synthetic *pgs*BCA genes are homologous to the *cap*BCA genes of *B. anthracis*. The study identified a *pgs*E subunit of *B. endophyticus,* which is analog to *cap*E in *B. anthracis* (Fig. 4) and also referred to *ywt*C in *B. subtilis*. The subunit *pgs*S (*ywt*D) is present in the *B. subtilis* and *B. licheniformis* PGA synthetic operon and absent from *B. endophyticus* and *B. anthracis* PGA synthetic operon (Fig. 4). The amino acid sequence of *B. endophyticus cap*C is 82% similar to *B. anthracis cap*C*,* indicating a high probability of *cap*C region primer annealing in either *B. endophyticus* or *B. anthracis* strains. The capsule regulons *acp*A and *acp*B in *B. anthracis* were observed on the same PGA operon. In *B. endophyticus* genomes, none of these two regulons were observed in the PGA complex operon (Fig. 4).

### Glutamyltranspeptidases (*ggt*)

**Fig. 5:**
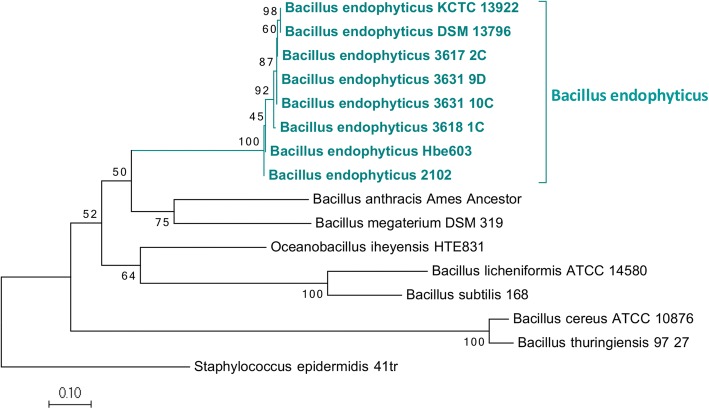
Maximum likelihood phylogenetic tree showing the relationship of the gamma-glutamyltranspeptidase (*ggt*) sequence of *Bacillus endophyticus* strains with related sequence strains of *Bacillus* species

An open reading frame (ORF) that encodes for *γ*-glutamyltranspeptidases (GGT) was present in the sequenced *B. endophyticus* strains (Fig. 4b) and other 4 compared *B. endophyticus* strains (2102, Hbe603, KCC 13922, DSM 13796). In this study, the nucleotide sequence analysis of the *ggt* in *B. endophyticus*, *B. anthracis* and other *Bacillus* species showed sequenced *B. endophyticus* strains cluster with the compared *B. endophyticus* strains (Fig. 5). Single nucleotide and amino acid variations were observed between aligned *ggt* of *B. endophyticus* and *B. anthracis* 20SD. The aligned *ggt* amino acid sequences of the reported *B. endophyticus* strains in this study are 44% identical to *B. anthracis* (Fig. 4). The sequenced *B. endophyticus* strains in the study had the same nucleotide identity profile with *B. endophyticus* DSM 13976 and KCTC 13922. *B. endophyticus* 3618_1C grouped separately amongst the other *B. endophyticus* strains, and this was also observed in the heat map (Fig. 2). There was a clear separation between the *ggt* of the *B. endophyticus* strains and the other *Bacillus* species, with the closest being *B. anthracis* Ames ancestor and *B. megaterium* (Fig. 4).

### *Bacillus endophyticus* and *B. anthracis* features

The annotation of *B. endophyticus* strains and *B. anthracis* showed the presence of the import and iron release four-gene cluster (feuABCD) and the Fe-bacillibactin (iron carrier) uptake system common in both. The four-gene operon of *feu*A*-feu*B*-feu*C*-feu*D and trilactone hydrolase (bacillibactin) siderophore *YuiI* (*Bes*A) was identified in the *B. endophyticus* genomes. Bacillibactin siderosphore is synthesized through the alternative non-ribosomal peptide synthetase pathways and helps the bacterium in iron acquisition from their environment [21]. Genes identified in both *B. endophyticus* and *B. anthracis* also included bacitracin ABC transporters, bacitracin export ATP-binding protein *Bce*A and permease protein *Bce*B, which confers resistance to bacitracin or stress response as defensive mechanisms.

## Discussion

The presence of the PGA subunits *pg*s/*cap*A-C in the South African *B. endophyticus* strains isolated alongside *B. anthracis* strains from the 2009 anthrax outbreak initiated the comparative investigation of these two species. *B. endophyticus* and *B. anthracis* can be differentiated based on sensitivity to the γ-phage [13], which is not a reliable differentiating character as resistance to γ-phage had been reported amongst the normally γ-phage sensitive *B. anthracis* [13, 22]*.* In this study a more comprehensive approach that included morphology, biochemical as well as WGS were used to compare these two species in order to identify differentiating characteristics for diagnostic purposes. *B. endophyticus* has not been reported to date to be isolated with *B. anthracis.* This and the presence of PGA genes in *B. endophyticus* is noteworthy since capsule genes is an important diagnostic characteristic of *B. anthracis*. *B. anthracis* could be differentiated from *B. endophyticus* based on unique capsulated box-shaped bacilli in long chains (in culture), γ-phage susceptibility characteristic and the presence of the toxin *pag* gene. *B. endophyticus* showed round-edged bacilli present either as single cells or in short chains, γ-phage resistant and the absences of the toxin *pag* gene. Identification of the *pgs*/*cap*BCA genes of the PGA biosynthetic pathways in both species using WGS comparative analysis shows the value of this approach*.* The *pgs*BCA, γ-glutamyl-transpeptidase (*ggt*) and *pgs*E open reading frames were identified in the chromosomes of *B. endophyticus* genomes.

The South African *B. endophyticus* strains were differentiated from *B. anthracis* based on γ-phage microbiological characteristics and real-time PCR, whereas 16S rRNA sequences and Omnilog identified the *B. endophyticus* strains [13]. However identification of the *B. cereus sensu lato* group using 16S rRNA gene sequencing is often challenging, since it has been regarded as a single taxon based on similar 16Sr RNA sequences [23]. The diagnosis of *B. anthracis* require the use of microbiology characteristics as well as conventional or real-time PCR that detects *B. anthracis* specific chromosomal regions, toxin genes on pXO1 and capsule genes on pXO2 [2]. However, regions similar to the *B. anthracis* plasmids (pXO1 and pXO2) have been reported in other *Bacillus* species [11, 12] as observed with conventional PCR of *B. endophyticus* that amplified *cap*A, *cap*B and *cap*C regions [13].

Previous studies have reported a close relationship between *B. endophyticus* and *B. smithii* [1], which was also demonstrated in this study (Table 2, Additional file 3: Figure S3). They could be differentiated based on capsule, motility and rods morphology appearance [1, 13, 24]. The WGS of *B. endophyticus* strains reported in the study were closely related to *B. megaterium* DSM 319 using RAST as reported in *B. endophyticus* 2102 WGS [25]. However, *B. megaterium* DSM 319 does not contain any plasmids unlike other *B. megaterium* strains [26] and this has the potential to create a bias in RAST annotations [17]. *B. megaterium* bacilli (2.0–5.0 μm) are slightly larger than *B. endophyticus* (2.5–3.5 μm) and both are non-motile (Table 2). Features of *B. megaterium* can be confused with *B. anthracis* as both are non-motile, encapsulated and some *B. megaterium* strains are non-heamolytic [27], but can be differentiated based on penicillin and γ-phage sensitivity [28]. The γ-phage sensitivity is noted in *B. anthracis* strains containing the γ-phage receptor GamR gene [29]. None of the sequenced and compared *B. endophyticus* genomes had this gene. *B. endophyticus* is also non-motile, non-hemolytic, and penicillin sensitive, which do not distinguish it from *B. anthracis*. *B. megaterium, B. endophyticus* and *B. anthracis* can be differentiated based on morphology followed by verification of virulence factors and/or prophage region using real-time PCR [30].

None of the lambda prophage regions of *B. anthracis* were found in *B. endophyticus* using WGS comparative analysis. As indicated the prophage regions of *B. anthracis* lambdaBa03 (01–04) accurately distinguished *B. anthracis* from *B. endophyticus* and other related *Bacillus* species [30]. The *B. endophyticus* strains in this study presented many different prophage regions. The *B. endophyticus* strains 3618_1C shared common prophages with the *B. endophyticus* DSM_13,796 and KCTC 13922. Jia et al. [16] determined four prophage regions in the *B. endophyticus* Hbe603 strain, which were determined as hypothetical proteins that are different from the prophages in *B. endophyticus* strains reported in this study. The shared prophage regions amongst *B. endophyticus* strains can be investigated as more genomes become available that could be used in diagnostic assays.

WGS of the sequenced *B. endophyticus* strains in this study are closely related to *B. endophyticus* DSM 13796 and KCTC 13922 based on the average nucleotide identify (Fig. 2). The overrepresentation of the COG in the core cluster analysis might show that *B. endophyticus* has a high number of highly conserved genes and that horizontal gene transfer does not necessarily play a major role in its evolution*.* One key feature of *B. endophyticus* identified through WGS is the bacillibactin-associated genes for biosynthesis that are also present in *B. anthracis* and many other members of the *B. cereus sensu lato* group [21]. The bacitracin cluster of genes identified in *B. endophyticus* and *B. anthracis* are known to be a peptide antibiotic that is non-ribosomally synthesized in some strains of *Bacillus* [31], especially in *B. subtilis*. It has the ability to disrupt the cell wall and peptidoglycan synthesis of the gram-positive and gram-negative bacteria. However, bacillibactin and bacitracin cannot be used as differentiating features of *B. endophyticus* strains since they are also present in *B. anthracis* strains.

*B. endophyticus* Hbe603 consists of one chromosome and 8 plasmids that belong to the members of *Bacillus* group [16]. The function or the role of the plasmids has not yet been studied. Sequence comparison revealed no similarities between *B. endophyticus* and *B. anthracis* plasmids. The PGA complex is present in most *Bacillus* species including *B. licheniformis* [32]*, B. subtilis* [10], *B. anthracis* [4] and *B. cereus sense lato* group including *B. cereus* biovar *anthracis* [12, 33]. In this study, the PGA biosynthesis operon was also identified in the *B. endophyticus* genomes. The PGA subunits are located in the chromosome of the *B. endophyticus* strains unlike in the plasmid of *B. anthracis*.

The polyglutamate depolymerase *cap*D is present in the *B. anthracis* [7] and belongs to the γ-glutamyltransferase (GGT) family. This gene is responsible for the covalent anchoring of the capsule to peptidoglycan and act as a depolymerase in *B. anthracis* [7]. *B. anthracis cap*D gene is related to *B. subtilis* natto *ywr*D and *B. licheniformis* DSM13 *ggt*. However, *ywr*D or *ggt* is located in the chromosome and reside in a locus distant from the *pgs*BCA subunits. The *ggt* and *cap*D subunits were present in both *B. endophyticu*s and *B. anthracis* genomes respectively (Fig. 4). The *ggt* is located on a locus adjacent to the pgs*BCA* subunit genes in the chromosome of the sequenced *B. endophyticus* (3631_9D, 3618_1C, 3631_10C, 3617_2C) and other compared *B. endophyticus* genomes (2102, Hbe603, KCC 13922, DSM 13796). The *ggt* identified in the *B. endophyticus* has different nucleotide and amino acid variations with *B. anthracis* and *B. subtilis*. Annotation of this subunit in *B. endophyticus* strains showed that is not linked with the attachment of the PGA to peptidoglycan, however it’s associated with the PGA biosynthesis. The identified γ-glutamyltransferase in *B. endophyticus* genomes may suggest that it hydrolyses PGA biosynthesis as suggested for *B. subtilis ggt* that hydrolyses the PGA in an exo-type manner [34]. In *B. subtilis* NAFM5, the GGT was shown to have hydrolysed γ-D-L PGA from the D- and L-glutamate during stationary phase through transcriptional activation [35].

The *pgs*E subunit is known to stimulate the PGA production in the presence of zinc [4]. However in *B. subtilis*, high concentrations of *pgs*B, *pgs*C, and *pgs*A were determined to form PGA in the absence of *pgs*E [36]. There is a small ORF present in *B. endophyticus* (Fig. 4) strains annotated as hypothetical protein, which has the same nucleotide size (144 bp) than *B. anthracis cap*E. Protein alignment of *B. endophyticus pgs*/*cap*E is 42% identical to *cap*E of *B. anthracis*. This ORF may be important for the PGA biosynthesis and act as an anolog *pgs*/*cap*E since *B. anthracis cap*E is required for PGA biosynthesis [4]. The small ORF is found after the *ggt*/*cap*D in both the *B. endophyticus* and *B. anthracis* PGA operon (Fig. 4)*.* The *B. subtilis pgs*S is an exo-γ-glutamyl hydrolase that is linked to the release of PGA in the environment [4]. The γ-D-L-glutamyl hydrolase *pgs*S lies immediately downstream of *pgs*BCA genes in *B. subtilis* chromosome [37]. This subunit encode enzyme that cleaves the glutamyl bond between D- and L-glutamic acids of PGA. The *pgs*S subunit was not identified in the *B. endophyticus* genomes. An ORF was identified in the PGA operon of *B. endophyticus* genomes, annotated as a putative esterase/lipase, that lies immediately downstream after the *pgs*E. This putative extracellular esterase belongs to the hydrolase enzymes family that might also be involved in the hydrolysis of PGA, but this hypothesis needs further investigation. The regulatory genes, *acp*A, *acp*B and *atx*A (located in pXO1) are known to control the expression of *B. anthracis* capsule PGA biosynthesis operon *cap*BCADE [5]. The two regulons *acp*A and *acp*B located in the pXO2 were observed in the *B. anthracis* 20SD PGA biosynthesis operon, which is absent in the *B. endophyticus* PGA operon.

The exo-polysaccharide biosynthesis ORF was identified in the *B. endophyticus* genomes. It consisted of manganese-dependent protein-tyrosine phosphatase, tyrosine-protein kinase transmembrane modulator *eps*C, and tyrosine-protein kinase *eps*D. The tyrosine-protein kinase transmembrane modulator *Eps*C and tyrosine-protein kinase *Eps*D are found in the same operon. Extracellular polysaccharides (EPS) are polymers that consist of different simple sugars. They are produced by variety of bacteria and may be assembled as capsular polysaccharides (CPS) tightly associated with the cell surface or they may be liberated into the growth medium. In *E. coli* and *B. subtilis* the *eps*C and *eps*D are reported to control UDP-glucose dehydrogenase activity [38, 39]. In *B. subtilis* strains, cells are held together by EPS and amyloid-like fibers for biofilm formation [40]. In *B. endophyticus* genomes, in the same operon of exo-polysaccharide, the UDP-glucose dehydrogenase and hyaluronan synthase enzymes were identified. Hyaluronan synthase is membrane bound enzyme that is used to produce the glycosaminoglycan hyaluronan at the cell surface through the membrane. Hyaluronan synthesis in most bacteria is associated with protecting the bacteria against host and environmental factors, which may be detrimental to survival [41]. The hyaluronic acid polysaccharide capsule was found in the *Streptococcus pyrogenes* [41]. In order for *S. pyrogenes* to synthesize a HA capsule, at least three different genes must be present and arranged in an operon designated the HA synthesis manner [41]. This includes the HA synthase and two sugar precursors (UDP-glucose dehydrogenase and UDP-glucose-pyrophosphorylase). In *B. endophyticus* genomes only one sugar precursor UDP-glucose dehydrogenase, and the hyaluronan synthase are present. The role of HA needs further investigation in the *B. endophyticus* strains.

## Conclusion

*B. endophyticus* is a gram-positive, non-motile, non-hemolytic, rod-shaped bacterium which is endospore forming, penicillin sensitive but γ-phage resistant. *B. anthracis* has all these characteristics in common with *B. endophyticus* with the exception that it is γ-phage sensitive bacterium. *Bacillus* species, which include *B. anthracis*, *B. megaterium*, *B. endophyticus* and *B. smithii* can be differentiated based on their morphological appearances and other microbiological features. However, most of these microbiological features (biochemical tests i.e. the presence of lecitinase, starch, VP test motility and other tests) are not routinely used for identification and characterization of *Bacillus* species. Molecular techniques such as real-time PCR targeting species-specific chromosomal markers, virulence genes and 16S rRNA sequencing, should continuously be used to identify or distinguish related *Bacillus* species. This can further be supplemented with specific prophages of the bacterium or other specific genes present in the genome. *B. endophyticus* is considered as industrial important due to biotechnology properties like the production of antibiotics such as fosfomycin and bacitracin.

*B. endophyticus* can easily be differentiated from *B. anthracis* based on the morphology appearance but confirmation of virulence factors like capsule genes identified in *B. endophyticus* could complicate anthrax diagnostics. Whole genome sequencing identified and differentiated *B. anthracis* and *B. endophyticus* PGA capsule genes. *B. anthracis* and *B. endophyticus* PGA biosynthesis subunits were determined to be located in the pXO2 and chromosome respectively. The *B. endophyticus* strains couldn’t synthesize a surface associated γ-PGA, suggesting that PGA helps the bacteria to survive under adverse conditions. Therefore *B. endophyticus* is a non-capsulated bacterium that survives at high salt concentrations. Prophage regions have emerged as key markers in distinguishing *B. anthracis* and eliminating other related *Bacillus* species. The study highlights the significance of using whole genome shotgun sequencing to identify virulence and other important genes that might be present amongst unknown samples from natural outbreaks.

## Methods

### Isolates

The *B. endophyticus* and *B. anthracis* isolates included in this study were isolates collected during the 2009 anthrax outbreak in the Northern Cape Province (NCP) of South Africa. These isolates included a *B. endophyticus* and *B. anthracis* isolate from the same animal. The *B. endophyticus* were isolated from blood collected from animal carcasses whereas *B. anthracis* isolates were isolated from soil below the carcass as well as blood collected from animal carcasses (Table 1). The *B. endophyticus* isolates exhibited some similar phenotypic and genetic similarities to those of *B. anthracis* [13] and therefore we characterized these isolates to enhance and contribute to the diagnosis of *B. anthracis*. The incubation condition for *B. endophyticus* range from 10 to 55 °C although the optimum growth temperature is between 28 and 30 °C, but this study used conditions specific for anthrax diagnosis as described by the International protocols for anthrax [42].

### Phenotypic characterization

In this study we focused mainly on capsule characterization of *B. endophyticus* strains to enhance the phenotypic characterization previously done on South African *B. endophyticus* and *B. anthracis* stains [13] as well as summarizing phenotypic characterizations of related *Bacillus* species. Four *B. endophyticus* and three *B. anthracis* strains isolated from animal anthrax cases in NCP in South Africa available at Agricultural Research Council–Onderstepoort Veterinary Institute (ARC-OVI) were used in this study (Table 1). The *B. endophyticus* and *B. anthracis* isolates were collected from 2009 anthrax outbreaks in the NCP of South Africa (Table 1). The samples were processed at the ARC-OVI reference laboratory (Onderstepoort, South Africa), where *B. anthracis* suspected cases are confirmed. Pure cultures were grown on 5% SBTA, followed by incubation at 37 °C for 24 h for the observation of colony morphology and to determine hemolytic activity [42]. Colony morphology was observed on nutrient agar containing 0.8% sodium bicarbonate following incubation in the presence of 5% CO_2_ at 37 °C for 24–48 h in the dark to induce capsule formation. The capsules from strains incubated on 0.8% sodium bicarbonate supplemented nutrient agar were stained using India ink, Giemsa and copper sulphate followed by visualization using light microscopy [42, 43]. Each culture was also transferred to blood serum and incubated under both aerobic and anaerobic conditions at 37 °C for 24 h to determine the formation of a capsule [42]. Blood smears were stained using Rapi-Diff and visualized by light microscopy. The positive control for the capsule production included *B. anthracis* 3618_2D (cap^+^, virulent strain) [13] whereas the negative controls included *B. licherniformis* ATCC 12759 (cap^−^) and *B. anthracis* Sterne (cap^−^) strains. Phenotypic properties of *B. endophyticus* and *B. anthracis* were compared with those from published literature including *B. megaterium* and *B. cereus* as shown in Table 2 ([1, 24, 27, 42, 44], http://www.tgw1916.net).

### Genomic DNA extraction

*B. endophyticus* and *B. anthracis* strains (Table 1) were inoculated in 2 ml nutrient broth, followed by overnight incubation at 37 °C. The cells were harvested by centrifugation at 5000 xg for 10 min. Genomic DNA was extracted from the harvested cells using the DNAeasy Tissue kit (Qiagen, Germany) according to the manufacturer’s instructions. The isolated DNA was then quantified using the Qubit® fluorometric method (Life Technologies, USA) according to the manufacturer’s instructions. The DNA integrity was monitored through electrophoreses using a 0.8% agarose gel pre-stained with ethidium bromide and visualized on a UV transilluminator.

### High-throughput sequencing

Shotgun library preparation of four *B. endophyticus* (Table 1) strains was performed using the Nextera DNA Sample Preparation kit (Illumina, USA). Clusters generation and the sequencing were performed using the TruSeq™ PE Cluster kit v2-cBot-HS and TruSeq SBS v3-HS (200 cycle) kit respectively (Ilumina, USA). The sequencing was performed on the HiScan SQ sequencer (Illumina, USA).

### Genome assembly and annotation

Sequence data quality was assessed using FastQC software v 0:10.1 [45]. Ambiguous nucleotide sequence and sequence adapters were trimmed using CLC Genomic Workbench 7.5 (Denmark). The de novo assemblies were performed using the CLC Genomic Workbench 7.5. The *B. endophyticus* strains contigs were further extracted and analyzed with BLASTn [46] using *B. endophyticus* Hbe603 (Genbank accession no: CP011974) as reference genome. MAUVE tool [47] was used to order the sequence of *B. endophyticus* reported in the study using *B. endophyticus* Hbe603 as a reference. The assembled contigs were annotated using NCBI prokaryotic genome automatic annotation pipeline (PGAAP) and rapid annotation using subsystem technology [48] annotation server for subsystems and functional annotation [17]. The presence of prophage sequence regions in the 8 *B. endophyticus* genomes (3631_9D, 3631_10C, 3618_1C, 3617_2C, Hbe603, 2102, KCC 13922, and DSM 13796) were determined using PHAge Search Tool (PHAST) [49].

### 16S rRNA gene phylogenetic analysis

The 16S rRNA sequence region consisting of approximately 1500 bases was extracted from the assembled genomes of *B. endophyticus* strains (3631_9D, 3618_1C, 3631_10C and 3617_2C). These sequences were further aligned and compared with the 16S rRNA gene sequences of *Bacillus* species available in NCBI (http:www.ncbi.nlm.nih.gov). NCBI BLAST homology searches of the 16S rRNA gene sequences were performed to assess homologous hits to sequences available in NCBI. Multiple alignments of the gene sequences extracted from assembled genomes and from those mined from NCBI were performed using MAFFT [50]. Maximum likelihood analysis of the *B. endophyticus* 16S rRNA nucleotide sequences and related *Bacillus* group sequences were performed using 1000 bootstrap iterations in MEGA 6.0.

### Average nucleotide identities, pan-genome analyses and functional classification of orthologous genes

The CDSs (coding domain sequences) of *B. endophyticus* sequenced strains were subsequently compared against each using pair-wise BLASTn, to allow for calculations of average nucleotide identities. The pan-genome homology of all the 8 *B. endophyticus* (3631_9D, 3631_10C, 3618_1C, 3617_2C, Hbe603, 2102, KCC 13922, and DSM 13796) were computed using the get homologues tool [51] with default parameters. In brief, the tool conducted similarity searches between the CDSs of all 8 genomes using pair-wise BLASTp [46], and these were subsequently clustered into the different pan-genomic categories using OrthoMCL [52]. The analysis resulted in four clusters, and these were defined as: core-genes present in all genomes; softcore-genes present in 95% of the genomes; shell-genes present in few but not all genomes; and the cloud – genes present in two or less of the genomes. The core and the softcore represent sets of conserved or house keeping genes. The softcore clusters were included in the analysis because the sequenced draft genomes of *B. endophyticus* strains in this study might be missing some of the essential genes. Both the shell and the cloud compose of accessory genes that play a role towards the lifestyle and adaptation characteristics of an organism to its particular environment.

The four clusters determined for the 8 genomes were searched for shared pattern similarities against a conserved domain database of cluster of orthologous groups using rps-blast with –E < 1e-3. Genes with shared pattern similarities were assigned classes that were later categorized into the COG (clusters of orthologous group) subgroups to determine their distributions for all the clusters.

### Polyglutamate (PGA) subunit genes analysis

The presences of the PGA synthesis genes were determined for the 8 *B. endophyticus* strains (3631_9D, 3631_10C, 3618_1C, 3617_2C, Hbe603, 2102, KCC 13922, and DSM 13796) using analyses on the RAST server with the annotated draft genomes [17]. The PGA-capsule subunits were extracted from the annotated contigs of the *B. endophyticus* genomes using comparative analysis of RAST. *B. anthracis* PGA capsule subunits were compared with the *B. endophyticus* PGA subunits using the same annotation system. BLASTp [46] was used to compare the PGA proteins of *B. anthracis*, *B. endophyticus* and *B. subtilis*. Phylogenetic tree analysis of the subunit gene *cap*D/*pgs*D of *B. endophyticus*, *B. anthracis* and other closely related species was constructed using maximum-likelihood. Multiple alignments of the gene sequences were constructed using multiple sequence alignment based on fast fourier (MAFFT) [50]. Alignment of the corresponding amino acid sequences was performed using CLC Genomic Workbench 7.5. MEGA 6.0 was used to construct the phylogenetic tree using 1000 bootstrap iterations.

### Genome sequences and accession numbers

The four sequenced genomes of *B. endophyticus* were deposited in the Genbank genome database under accession numbers: *B. endophyticus* 3631_9D LVYL00000000, *B. endophyticus* 3631_10C LVYK00000000, *B. endophyticus* 3618_1C LWAI00000000 and *B. endophyticus* 3617_2C LWAG00000000. The additional four genomes that were used in the comparative analysis of *B. endophyticus* strains were retrieved from NCBI genebank. Accession numbers: *B. endophyticus* Hbe603 GCA_000972245.3, *B. endophyticus* 2102 GCA_000283255.1, *B. endophyticus* DSM_13,796 GCA_900115845.1 and *B. endophyticus* KCTC 13922 GCA_001590825.1. The sequenced *B. endophyticus* genome sequences in the study were further compared to the South African *B. anthracis* 20SD and 3631_1C strains (Genbank accession nr LGCC00000000 and LGCD00000000).

## Additional files

Additional file 1: **Figure S1.** (1) Colony morphology of (a) *Bacillus endophyticus* that is small circular, wet and non-mucoid and (b) *B. anthracis* appear circular, mucoid on nutrient agar supplemented with sodium bicarbonate at 5% CO_2_ after incubation at 37 °C.  Colony morphology of *B. endophyticus* and *B. anthracis* on sheep blood agar incubated at 37 °C. *B. anthracis* shows the characteristic shiny, rough with ground-glass appearance compared to the white slimy and smooth colonies of *B. endophyticus*. (TIFF 2652 kb)

Additional file 2: **Figure S2.** Phenotypic electron microscopic examination of the morphology of *B. endophyticus* strains after 24 h incubation on nutrient agar containing 0.8% sodium bicarbonate stained using copper sulphate. (TIFF 4206 kb)

Additional file 3: **Figure S3.** Phylogenetic tree of 16S ribosomal RNA sequence of the *Bacillus endophyticus* 3618_1C, 3631_9D, 3617_2C and 3631_10C strains with related *Bacillus* species using maximum likelihood. *Geobacillus thermoglucosidasius* was used as an out-group. Bootstrap values > 60 are indicated at the internodes. (TIFF 219 kb)

Additional file 4: **Table S1.** Plasmid comparison of the four sequenced *Bacillus endophyticus* strains (3618_1C, 3631_9D, 3631_10C, 3617_2C) with *B. endophyticus* Hbe603 strain. (DOC 18 kb)

## Abbreviations

ANI: Average nucleotide identity
ARC-OVI: Agricultural Research Council–Onderstepoort Veterinary Institute
BLAST: Basic local alignment search tools
BLASTn: Basic local alignment search tool nucloetide
BLASTp: Basic local alignment search tool protein
CAP: Capsule
CDSs: Coding domain sequences
COG: Clusters of orthologous group
CPS: Capsular polysaccharides
EPS: Extracellular polysaccharides
GGT-gamma: Glutamyltranspeptidases
MAFFT: Multiple sequence alignment based on fast fourier
MAUVE: Multiple alignment of conserved genomic sequence with rearrangements
NA: Not available
NaCl: Sodium chloride
NCBI: National centre for biotechnology information
NCP: Northern Cape province
NGS: Next generation sequencing
ORF: Open reading frame
PCR: Polymerase chain reaction
PGA: Polyglutamte
PGAAP: Prokaryotic genome automatic annotation pipeline
PGS: Polyglutamate synthase
RAST: Rapid annotations using subsystems technology
WGS: Whole genome sequencing
*γ*: Gamma
