# Supplementary figures and images for: Whole genome sequencing and identification of Bacillus endophyticus and B. anthracis isolated from anthrax outbreaks in South Africa
Source: BMC Microbiol. 2018 Jul 9;18:67. doi: 10.1186/s12866-018-1205-9 (PMC6038202; doi:10.1186/s12866-018-1205-9)

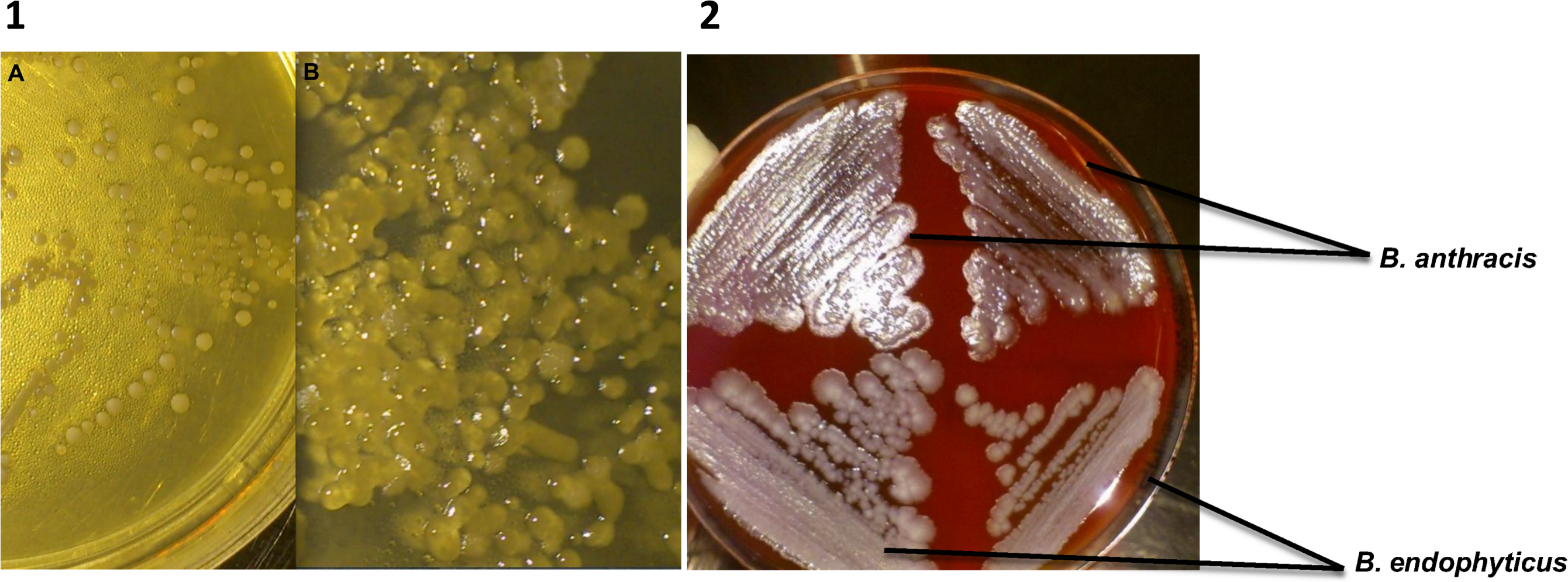

Supplement: Supplementary file 1 — Figure S1. (1) Colony morphology of (a) Bacillus endophyticus that is small circular, wet and non-mucoid and (b) B. anthracis appear circular, mucoid on nutrient agar supplemented with sodium bicarbonate at 5% CO2 after incubation at 37 °C. Colony morphology of B. endophyticus and B. anthracis on sheep blood agar incubated at 37 °C. B. anthracis shows the characteristic shiny, rough with ground-glass appearance compared to the white slimy and smooth colonies of B. endophyticus. (TIFF 2652 kb) [file 12866_2018_1205_MOESM1_ESM.tiff]

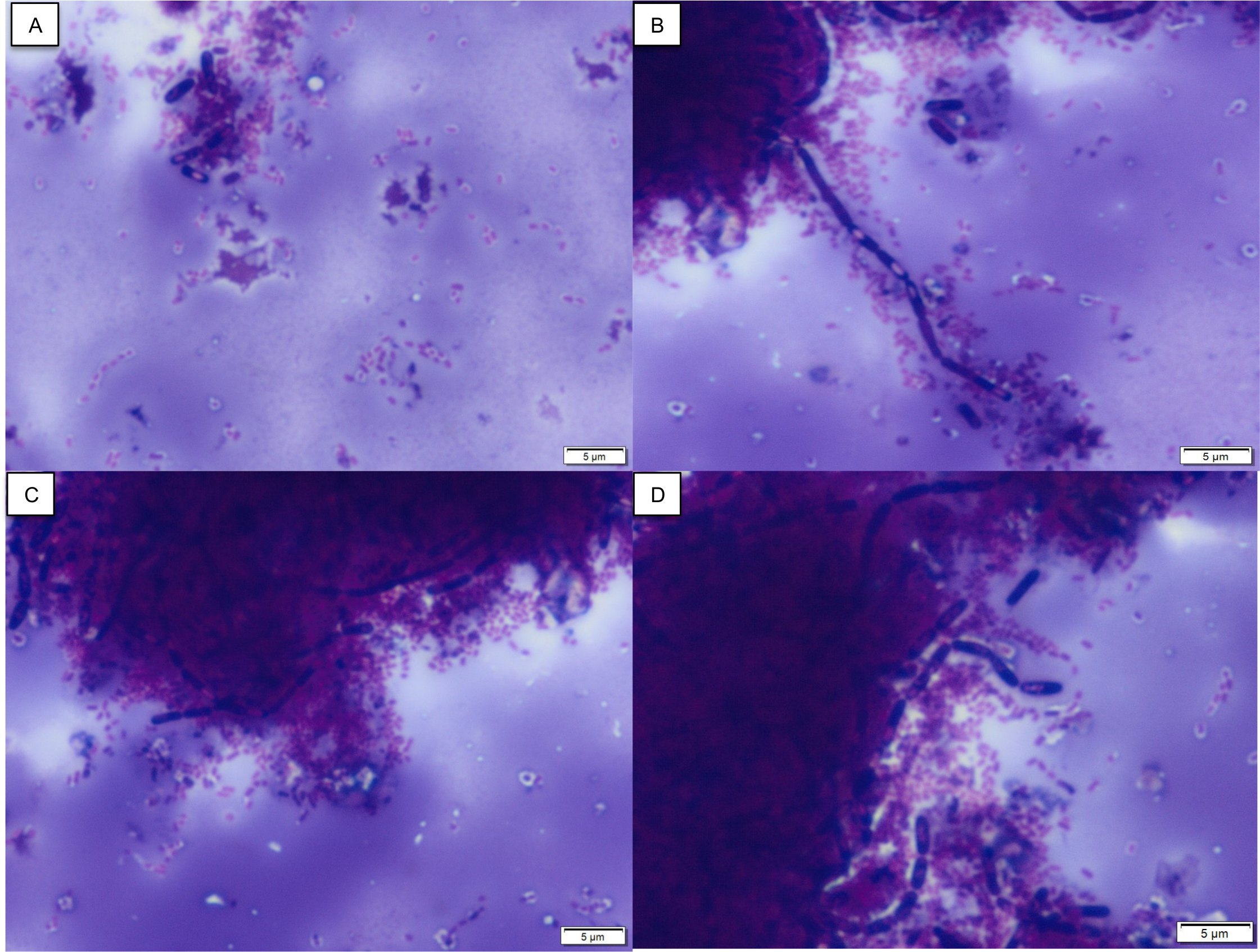

Supplement: Supplementary file 2 — Figure S2. Phenotypic electron microscopic examination of the morphology of B. endophyticus strains after 24 h incubation on nutrient agar containing 0.8% sodium bicarbonate stained using copper sulphate. (TIFF 4206 kb) [file 12866_2018_1205_MOESM2_ESM.tiff]

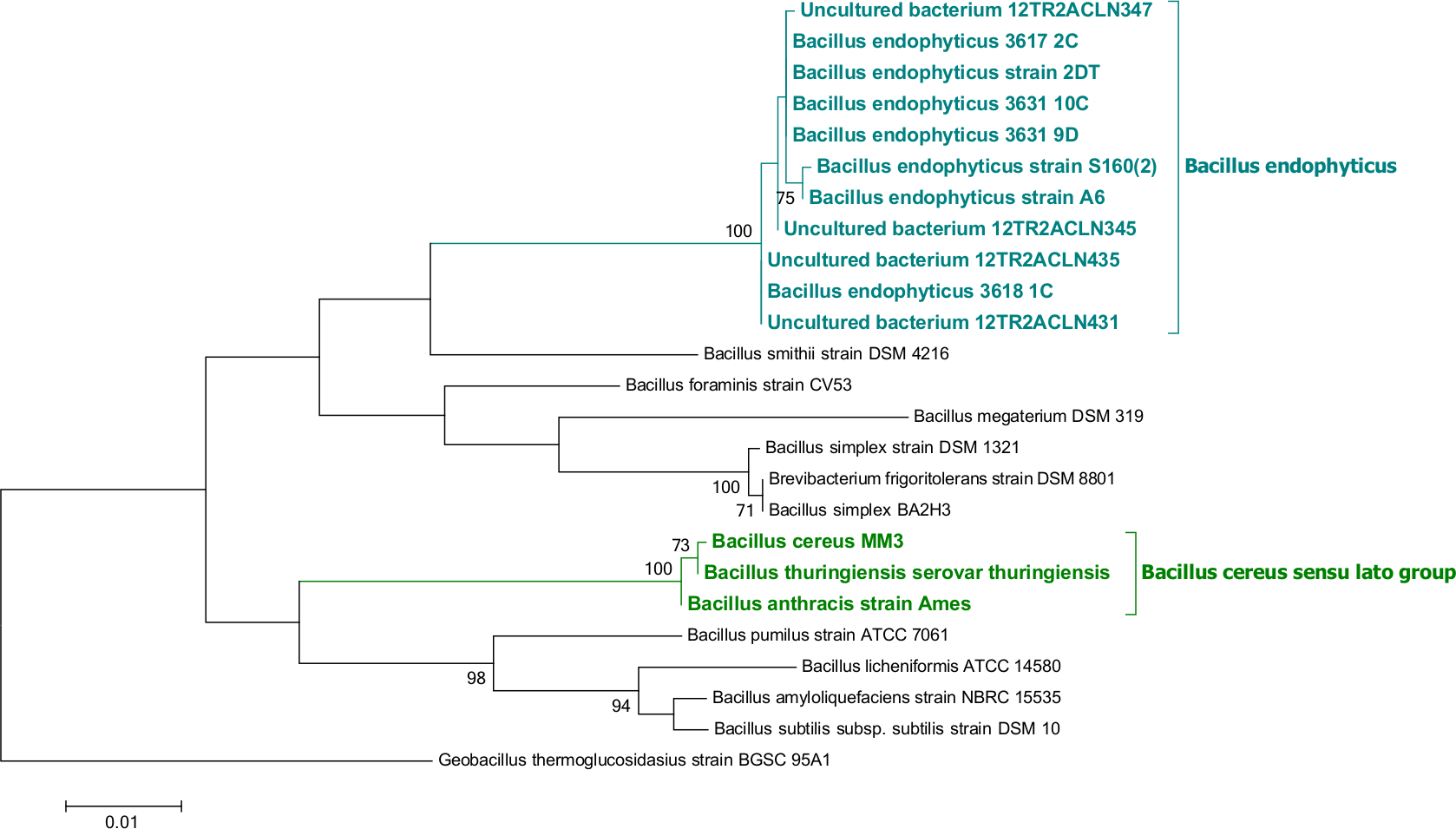

Supplement: Supplementary file 3 — Figure S3. Phylogenetic tree of 16S ribosomal RNA sequence of the Bacillus endophyticus 3618_1C, 3631_9D, 3617_2C and 3631_10C strains with related Bacillus species using maximum likelihood. Geobacillus thermoglucosidasius was used as an out-group. Bootstrap values > 60 are indicated at the internodes. (TIFF 219 kb) [file 12866_2018_1205_MOESM3_ESM.tiff]
