# Supplementary material for: Whole genome sequencing and identification of Bacillus endophyticus and B. anthracis isolated from anthrax outbreaks in South Africa
Source: BMC Microbiol. 2018 Jul 9;18:67. doi: 10.1186/s12866-018-1205-9 (PMC6038202; doi:10.1186/s12866-018-1205-9)
Supplement: Supplementary file 4 — Table S1. Plasmid comparison of the four sequenced Bacillus endophyticus strains (3618_1C, 3631_9D, 3631_10C, 3617_2C) with B. endophyticus Hbe603 strain. (DOC 18 kb) [file 12866_2018_1205_MOESM4_ESM.doc]

| **Plasmids** | ***B. endophyticus***  **Hbe603** | ***B. endophyticus* 3618_1C** | ***B. endophyticus* 3631_9D** | ***B. endophyticus* 3631_10C** | ***B. endophyticus***  **3617_2C** |
| --- | --- | --- | --- | --- | --- |
| **(Sizes in bp)** | | | | | |
| pBEH1 | 95 077  58 079  84 656  69 170  56 750  69 165  10 461  5 053 | 53 218  -  -  -  -  2 304  12 242  6 100 | 11 085  36 204  69 638  45 324  9 136  74 825  10 336  - | 61 665  -  9 154  40 099  1 674  18 757  15 783  - | 38 108  -  10 462  3 825  1 128  16 153  9 109  - |
| pBEH2 |  |  |  |  |  |
| pBEH3 |  |  |  |  |  |
| pBEH4 |  |  |  |  |  |
| pBEH5 |  |  |  |  |  |
| pBEH6 |  |  |  |  |  |
| pBEH7 |  |  |  |  |  |
| pBEH8 |  |  |  |  |  |
|  |  |  |  |  |  |
| Total plasmids | 8 | 4 | 7 | 6 | 6 |

Table S1. Plasmid comparison of the four sequenced *Bacillus* endophyticus strains (3618_1C, 3631_9D, 3631_10C, 3617_2C) with *B. endophyticus* Hbe603 strain.
